# The Cost of Provisions in Institutions.—IV

**Published:** 1904-04-30

**Authors:** 


### THE COST OF PROVISIONS IN INSTITUTIONS.—IV.

By the Author of "Hospital Expenditure—The Commissariat."

(*Continued from page* 64.)

### ST. GEORGE'S HOSPITAL (351 Beds).

The number of beds available in this hospital is 351, but owing to the temporary closing of ten wards in the year 1902, to which in this article reference is chiefly made, the actual number of beds occupied averaged only 251. It is obvious that fluctuations of expenditure calculated on the basis of occupied beds are inevitable. The closing of wards is almost a normal process in hospital work, and in old buildings like those at St. George's a continual round of repainting, re-flooring, and repairing is in general progress quite outside ordinary cleaning operations. A glance at the subjoined table will show this feature of fluctuation:—

| Year | No. of occupied beds | Cost of provisions per occupied bed | Cost of provisions per available bed |
|---|---|---|---|
| | | £ | £ |
| 1902 ... | 251 | 37½ | 26½ |
| 1801 ... | 300 | 32 | 27 |
| 1900 ... | 297 | 30 | 25¼ |
| 1899 ... | 323 | 27¾ | 25¼ |
| 1898 ... | 308 | 28 | 24¾ |

It will be seen that the variation of cost for provisions, calculated on the capacity of the hospital at a fixed unit of 351 beds, swings between a maximum of £27 and a minimum of £24¾, the difference between maximum and minimum cost not exceeding £2¼. Calculated on the cost per occupied bed the difference between maximum and minimum is nearly £10.

One other calculation has been attempted. The patients' cost is more easily estimated and less liable to fluctuation than that of other inmates of the hospital. The number of patients boarded is accurately recorded from year to year. A careful estimate has shown that the cost of boarding each in-patient at St. George's works out at 5s. 8d. a week. We have taken a general average of £14 15s. per annum for each occupied bed, and having deducted this amount from the total cost of provisions we have worked out a yearly average, based on the entire number of beds in the hospital (351), of the cost of provisions per bed for inmates other than patients.

| Year | No. of occupied beds out of 351 available | Total cost of provisions | Cost of patients at £14 15s. a year | Average cost of provisions exclusive of patients | |
|---|---|---|---|---|---|
| | | | | Per available bed | Per occupied bed |
| | | £ | £ | £ | £ |
| 1902 ... | 251 | 9,439 | 3,702 | 16¼ | 22¾ |
| 1901 ... | 300 | 9,571 | 4,425 | 14½ | 17 |
| 1900 ... | 297 | 8,944 | 4,380 | 13 | 15¼ |
| 1899 ... | 323 | 8,928 | 4,764 | 11¾ | 13 |
| 1898 ... | 308 | 8,754 | 4,543 | 13 | 14½ |

It must not be on any account assumed that increased expenditure in the matter of provisions indicates extravagant management. Nothing could be more mischievous than such a supposition. A rise of £2,277 in salaries and wages between 1899 and 1902 points to a large increase in the staff, and it will be apparent from the figures above that the cost of provisioning the patients is less than half the cost of provisioning the hospital.

It is necessary for the purpose of calculating the cost of the patients, to base the averages on the number of the occupied beds. But in estimating the upkeep of the staff, the average cost to the hospital is more accurately set forth if the total number of beds available for patients be considered as the unit. It is this, provided that no wards be permanently closed, which must necessarily regulate the size of the staff. There will be less pressure in any ward where only half the beds are occupied, but the number of nurses and probationers, wardmaids and servants, cannot be proportionately decreased. The saving in the cost of provisions subsequent on a decrease in the number of occupied beds is very easy to reckon, amounting as it does to about £100 for every seven beds. It is easily overpowered by changes in the household, as will be seen by comparing the figures for 1899 with those for 1902. In the latter year the average number of patients was less by 72 than in the former; but instead of a saving of £900, there is an addition of over £500 to the cost of provisions. We must again protest against conclusions adverse to the domestic management being drawn from these figures. They form merely a powerful illustration of the fact to which we have often drawn attention, that the cost of the patients' food is not the most considerable strand in the hospital commissariat; and that hence calculations based on the number of occupied beds require to be supplemented by additional information.

The number of "persons other than patients" boarded at St. George's Hospital in 1902 was as follows:—

| | Number | Proportion of available beds to each | Occupied beds |
|---|---|---|---|
| Medical and other officers ... ... | 12½ | 28 | 19·3 |
| Nursing staff ... | 144 | 2·5 | 1·7 |
| Male servants ... | 3 | | — |
| Female servants and wardmaids ... | 53 | 6·6 | — |
| Total ... ... | 212½ | 1·6 | — |

If the number of occupied beds be taken for the year 1902 the analysis of the household shows 212½ persons other than patients, as compared with 251 patients. The percentage is 45 of the total inmates. But as the staff of the institution has to be maintained in accordance with its size, rather than with the number of patients accommodated at any given moment, the figures based on an average of 351 beds should be compared with the others.

Taking now the published accounts for the year 1902, it will be instructive for purposes of comparison to work out the average cost per head of patients and non-patients taken together under the different items enumerated.

Daily average of patients ... ... ... 251
Daily average of non-patients ... ... ... 213
Daily average boarded in hospital ... ... 464

| | Total cost | Cost per head all inmates |
|---|---|---|
| | £ | £ s. |
| Meat ... ... ... | 2,837 | 6 2 |
| Brand's essence, etc. ... | 46 | 0 4 (patients only) |
| Fish ... ... ... | 698 | 1 10 |
| Poultry ... ... ... | 260 | 0 11 |
| Butter, cheese, etc. ... | 1,133 | 2 8 |
| Eggs ... ... ... | 493 | 1 1 |
| Milk ... ... ... | 1,717 | 3 14 |
| Bread and flour ... ... | 557 | 1 4 |
| Grocery ... ... ... | 827 | 1 15 |
| Jelly ... ... ... | 78 | 0 6 (patients only) |
| Vegetables ... ... | 479 | 1 0 |
| Malt liquor ... ... | 338 | 0 14 |
| Ice and mineral waters... | 92 | 0 4 |
| Wines and spirits ... | 206 | 0 16 (patients only) |

In an average week the consumption of meat by the patients was 877 lb., including also meat for beef-tea and broth. Taking the number of patients at 251, this would allow an average of 3·1 lb. per head per week. Of this 238 lb. was gravy beef, and 159 lb. scrag of mutton—presumably for making broth or soup. In an average week there were 112·4 every day on ordinary diet, as follows:—

*Dinner.*

Men ... ... 4 oz. cooked meat } ½ lb. potatoes and

Women ... 3 oz. cooked meat } pudding.

*Supper.*

1 pint soup.

In addition to a daily allowance of ¼ oz. tea, 1 oz. sugar, ¼ pint milk, 1 oz. butter, bread at discretion.

In the same week the fish diets numbered 863, or a daily average of 123·2. Fish diet is as follows:—

*Dinner.*

4 oz. cooked fish, ½ lb. potatoes and pudding.

*Supper.*

1 pint of milk.
Daily allowance as above.

The fancy diets, including milk or fever diet, as well as those in which extras are included, numbered 593 in the average week, or a daily average of 84·7.

The cost of boarding the nursing staff is estimated at £17 14s. a year.

The cost of boarding the medical officers is estimated at about £60 a year each.

The cost of boarding the patients is 5s. 8d. a week, or £14 15s. a year.

It is sufficient to add that a very high standard of quality is maintained in the provisioning of the hospital throughout.

